# Fetal Undernutrition Programming, Sympathetic Nerve Activity, and Arterial Hypertension Development

**DOI:** 10.3389/fphys.2021.704819

**Published:** 2021-11-17

**Authors:** Vinícius Schiavinatto Mariano, Patrícia Aline Boer, José Antônio Rocha Gontijo

**Affiliations:** Fetal Programming and Hydroelectrolyte Metabolism Laboratory, Nucleus of Medicine and Experimental Surgery, Department of Internal Medicine, Faculty of Medical Sciences, State University of Campinas, São Paulo, Brazil

**Keywords:** maternal low-protein diet, catecholamines, fetal programming, sympathetic nervous system, arterial hypertension

## Abstract

A wealth of evidence showed that low birth weight is associated with environmental disruption during gestation, triggering embryotic or fetal adaptations and increasing the susceptibility of progeny to non-communicable diseases, including metabolic and cardiovascular diseases, obesity, and arterial hypertension. In addition, dietary disturbance during pregnancy in animal models has highlighted mechanisms that involve the genesis of arterial hypertension, particularly severe maternal low-protein intake (LP). Functional studies demonstrated that maternal low-protein intake leads to the renal decrease of sodium excretion and the dysfunction of the renin-angiotensin-aldosterone system signaling of LP offspring. The antinatriuretic effect is accentuated by a reduced number of nephron units and glomerulosclerosis, which are critical in establishing arterial hypertension phenotype. Also, in this way, studies have shown that the overactivity of the central and peripheral sympathetic nervous system occurs due to reduced sensory (afferent) renal nerve activity. As a result of this reciprocal and abnormal renorenal reflex, there is an enhanced tubule sodium proximal sodium reabsorption, which, at least in part, contributes directly to arterial hypertension development in some of the programmed models. A recent study has observed that significant changes in adrenal medulla secretion could be involved in the pathophysiological process of increasing blood pressure. Thus, this review aims to compile studies that link the central and peripheral sympathetic system activity mechanisms on water and salt handle and blood pressure control in the maternal protein-restricted offspring. Besides, these pathophysiological mechanisms mainly may involve the modulation of neurokinins and catecholamines pathways.

## Background

The developmental origins of the health and disease hypothesis, which emphasizes environmental conditions before and immediately after birth, proposes that the gestational period significantly impacts the development, health, and well-being outcomes of an individual ranging from infancy to adult age. For years, the birth weight of a fetus has been demonstrated to depend on the maternal-fetal nutrient supply, placental blood flow, and maternal intrauterine environment (McCance, 1962). All these variables are involved in the onset of cardiovascular and metabolic disorders in adulthood. Three characteristics mark the fetal origin of diseases: latency, wherein effects may not be apparent until much later in life; persistency, whereby conditions resulting from a fetal development continue to exist for a given individual and progeny; and epigenetic effect, which describes the changes in phenotypic expression due to prenatal environment (Ashton, 2000). Two decades ago, Barker (1995) proposed that the maternal environment could induce adult diseases based on observed epidemiological associations between low birth weight and an increased risk for ischemic heart diseases and, particularly, arterial hypertension, renal and metabolic disorders, and hypertension (Wadsworth et al., 1985; Barker and Osmond, 1988; Barker et al., 1989; Barker, 1990, 1995, 2003; Barker and Martyn, 1992; Barker and Bagby, 2005). Although the relationship seemed variant, the bulk of evidence suggested a meaningful direct or indirect interaction between low birth weight and subsequent hypertension (Barker, 1995, 2003).

A systematic review from 80 studies observed that enhanced blood pressure (BP) occurred at a higher incidence in children and adults with low birth weight and reported that systolic BP is reduced by approximately 2 mmHg for every 1 kg increase in birth weight (Huxley et al., 2000, 2002; Lackland et al., 2000, 2002). Also, the study of Pima Indians patients with a history of maternal undernutrition and diabetes mellitus displayed an elevated risk of developing diabetic nephropathy (Nelson et al., 1998). Additionally, it has been demonstrated that maternal undernutrition is associated with more rapid progression of the kidney diseases, such as IgA nephropathy, membranous nephropathy, and minimal-change illness, suggesting that the kidneys of these infants are more vulnerable to future insults (Zidar et al., 1998).

Similarly, authors have found that imbalanced nutritional intakes are related to developing metabolic and cardiovascular diseases in offspring in adult life (Campbell et al., 1996; Roseboom et al., 2001). These studies established the concept of fetal programming, as described by Lucas (1991), as long-term changes in the structure and function of the organism of an individual that experienced insults during critical periods of development (Lucas, 1991; Barker, 1995). Additional supporting evidence showed that maternal environmental adversities directly modulate the embryotic or fetal development, impairing fetal growth and, consequently, the low weight at birth (Gluckman et al., 2005). The birthweight of a fetus depends on the maternal-fetal nutrient supply, placental blood flow, and maternal intrauterine environment (McCance, 1962; Ashton, 2000). Therefore, we may affirm that socioeconomic poverty and maternal undernutrition will reduce the nutrient supply to embryo-fetal development. Thus, factors that frustrate the passage of nutrients through the placenta, such as smoking, hypertension, and diabetes mellitus vessel disorders, are associated with an increased risk of developing the fetal origins of adult diseases.

Over the years, this aggregate of data and knowledge has made it possible to establish the causal conceptual construct about the fetal origins of adult disease. In theory, during development, organs and systems pass through a period of plasticity and vulnerability to the adverse changes of the environment, which leaves a durable imprint that affects subsequent health when natural postnatal conditions change. So, the phenotypic expression is matched to suboptimal intrauterine conditions, which impact fetal growth. These adaptive processes are aimed to increase the likelihood of survival *in utero* and after birth with expected maintenance of borderline or inadequate environmental conditions. However, this response may result in adverse long-term consequences in adulthood, especially when the postnatal environment affords more favorable growth conditions than those experienced *in utero*. Nevertheless, the strength of the association between the adverse intrauterine environment and subsequent arterial hypertension onset remains widely unclear once authors claim that the suggested association results from inappropriate adjustments for confounding factors (Huxley et al., 2002). Huxley et al. (2000, 2002) found a weak trend association between low birth weight and increased blood pressure in uneven and non-compared sample size studies (Gopalakrishnan et al., 2005). However, it seems that although the relationship is variant, the bulk of evidence suggests a crucial connection between birth mass and arterial hypertension development (Barker and Bagby, 2005).

In this way, epidemiologic data show that the high prevalence of arterial hypertension remains the leading risk factor for cardiovascular disease and death worldwide (Kearney et al., 2005; WHO, 2018). Heart disease and vascular injury are the most counted cause of non-communicable diseases (NCDs), which comprise the cause of 71% of death in the world population (WHO, 2018). Recent studies have shown that the number of hypertensive individuals increased substantially in the past decades, mainly in low and middle-income countries (Kearney et al., 2005; Mills et al., 2020). In these places, most of the three-quarters of NCD deaths occur (WHO, 2018). The associated etiological factor to the different types of NCDs depend on lifestyle and environmental factors including, e.g., nutrition, stress, physical activity, exposure to air pollution, and tobacco smoke, but the mechanisms by which this occurs remain unclear. Therefore, these insights also addressed early life as one of the critical periods that modulated the developmental susceptibility of these diseases and supported that low birth weight increases the risk of NCDs in later life along with a strong association between the increase of mortality for cardiovascular diseases and underweighting progeny (Barker et al., 1989, 2002a,2002b; Osmond et al., 1993; Gopalakrishnan et al., 2005; Fall, 2013). A hypertension family history is associated with an increased risk and earlier onset of hypertension in the offspring. Thus, primary hypertension development is a complex trait resulting from the interactions of multiple environmental exposures superimposed on an inherited genetic predisposition (Kunes and Zicha, 2009; Poulter et al., 2015). In humans and other mammals, highlighting the importance of congenital and hereditary aggregation of hypertension is well recognized (Lackland et al., 2000, 2002). Environmental, perinatal adverse, and lifestyle factors such as excess weight or obesity, physical inactivity, undernutrition, and excess sodium intake are associated with an increased risk of hypertension development. However, multiple renal, neural, endocrine, and cardiovascular control systems affect the heart and vascular homeostasis, making the pathophysiology of arterial hypertension extremely complex. Furthermore, the contribution of each of these factors to elevated arterial pressure is defined by gene-environment interactions or epigenetic phenotypic expression and varies among different individuals (do Carmo Pinho et al., 2003; Hall et al., 2012; Liang, 2018). Studies have demonstrated that gene variants and regulation of gene expression by epigenetic effects have an important influence on the risk of arterial hypertension development (Ehret and Caulfield, 2013; Friso et al., 2017). Multiple genetic analyses have uncovered several common variants of modest effect and low-frequency variants that contribute to hypertensive disease (Patel et al., 2017). Some large-scale genome-wide association studies have combined genetic information with biological insights into gene function, identifying variants at multiple loci associated with genes involved in arterial pressure homeostasis (Warren et al., 2017). Despite considerable advances in identifying genetic variants associated with a greater risk of hypertension development, it has been estimated that genetic risk scores obtained from a combination of multiple genetic variants associated with the risk of hypertension account for 3.5% of the trait variance (Dominiczak et al., 2017).

Thus, adequate analysis of the products of interactions between the gene expression and the environment in tissues relevant to the disease could be precious in overcoming these limitations. Unfortunately, environmental factors are difficult to quantify or even identify. Nevertheless, epigenetic deregulation has emerged as a hallmark of several complex pathologies, including metabolic disease and primary hypertension. Significant advances in our understanding of pathophysiological changes contribute to clarifying the complex origins of diseases in adult life, involving the deregulation of multiple control systems influencing blood pressure and the progression of end-organ damage related to high blood pressure impact. In this way, genome-wide association and replication study of blood pressure phenotypes among individuals (Hsu et al., 2003; Kato et al., 2007, 2011; Adeyemo et al., 2009; Cho et al., 2009) were performed in American, Asian, and African ancestry. This study revealed that single nucleotide polymorphisms are influencing arterial pressure firmly associated with methylation at multiple local CpG sites and microRNA expression (Kato et al., 2007, 2015; Sene et al., 2018, 2021), suggesting the presence of possible interactions between genomic and epigenetic regulation of blood pressure. In this sense, increasing evidence indicates that the origin of primary hypertension results from complex interactions between environmental and genetic factors, resulting in different risks and ages of onset of the disease within the general population.

The potential mechanisms of these alterations have emerged from the generation of animal models that mimic the effects of fetal programming. The most used models were designed through diet manipulations of the micro and macronutrient intake of pregnant animals, mainly manipulating the protein content of pregnant rodents, affecting fetal growth, renal development, and persistent hypertension in adulthood offspring (Langley-Evans et al., 1996, 1999; Langley-Evans, 2006, 2009). Prior studies have demonstrated the impact of severe gestational protein restriction in male rats on late life. These data showed that 6% low-protein restriction intake (LP) causes low birth weight and a 28% reduced number of nephrons. This effect is associated with decreased urinary sodium excretion and enhanced arterial blood pressure beyond 7 weeks of age in LP offspring compared with age-matched normal-protein intake (NP) (gestational 17% NP intake) offspring (Mesquita et al., 2010a,b; Sene et al., 2013). Studies have recently demonstrated that arterial pressure control changes are closely followed by renal glomeruli and tubular dysfunction of LP offspring. These effects could be, at least in part, attributed to renal sympathetic nervous system (SNS) action, precisely by the increase in renal sympathetic nerve activity (RSNA), which is increased is associated with reduced afferent (sensory) renal nerve response (DiBona, 2005; Custódio et al., 2017; Lamana et al., 2021). Here, we focus on highlighting studies supporting these findings and exploring the mechanisms involved in establishing arterial hypertension in maternal protein restriction progeny by altering sympathetic neural control, specifically in efferent and afferent renal nerve activities.

## Kidney Role on Arterial Pressure Development in Maternal Protein-Restricted Offspring

The human studies and experimental approaches conducted in models of intrauterine growth restriction (IUGR) and low birth weight concerning Barker’s hypothesis (Barker et al., 1989; Barker, 1990, 1998; Merlet-Benichou et al., 1994) have been implicated in fetal programming as a relevant causal factor to the origin of chronic renal dysfunction and cardiovascular disease in the adult life of experimental animals (Mesquita et al., 2010a,b; Sene et al., 2013; Lamana et al., 2021). Among the various critical situations to which the fetus could be exposed include maternal low-protein diet (Mesquita et al., 2010a,b; Sene et al., 2013), 50% (Vehaskari et al., 2001; Pinhal et al., 2013; Vaccari et al., 2015), and glucocorticoid exposure (Kvetnanský et al., 1993; Bertram et al., 2001; Jones et al., 2001). Many of these situations reflect those in which, unfortunately, human populations live in extreme poverty.

The role of the kidney in the pathogenesis of arterial hypertension has long been established. Recent studies, in the meantime, challenge the renal hegemony and suggest an essential change of the vascular cell abnormal response (Sampogna and Nigam, 2004; Crowley et al., 2005; Mendelsohn, 2005). However, this view has expanded and reviewed and now includes the concept of kidney dysfunction related to epigenetic events occurring during the perinatal period. Thus, reports have comprised the fetal programming changes in developing adult kidney disease and arterial hypertension (Sampogna and Nigam, 2004; Mesquita et al., 2010a,b; Sene et al., 2013, 2021; Lamana et al., 2021). This relationship between programmed fetal disease and kidney disorders involves several predisposing mechanisms, including impaired nephrogenesis, causing enhanced blood pressure during adulthood (Hinchliffe et al., 1991). The kidney is an organ centrally related to electrolyte balance and hemodynamic control. Notably, following cross transplants animal models, if the donor is a hypertensive subject, the recipient also develops arterial hypertension suggesting intrinsic renal factors involved in blood pressure control (Rettig et al., 1990; Guidi et al., 1996). Arterial hypertension development is closely linked to disorders of the glomerular filtration function, tubule sodium handling, and, consequently, in the extracellular composition changes of salt and fluid.

Confirming epidemiological data, experimental studies showed that inappropriate protein intake during pregnancy and lactation change the normal birth mass due to altering embryonic or fetal growth. Also, these reports showed a marked and sustained rise in arterial blood pressure associated with a decreased urinary sodium excretion in low-birthweight LP compared with age-matched NP offspring (Mesquita et al., 2010a,b; Lamana et al., 2021). In 2010, Mesquita and colleagues reported that, in male rat offspring whose mothers were submitted to severely protein-restricted (6% casein-diet) over entire pregnancy, there was an expressive reduction of the number of nephrons when compared to offspring from mothers kept on a normal-protein diet (17% casein) (Mesquita et al., 2010a,b). These studies also demonstrated that gestational embryonic and fetal protein restriction might lead to long-term renal sodium and water handle changes and structural alterations associated with progressive elevation of systemic blood pressure in male adult progeny (Brenner et al., 1998; Mesquita et al., 2010a,b; Pinhal et al., 2013; Sene et al., 2018, 2021; Vaccari et al., 2015; Lamana et al., 2021). Brenner and Chertow (1994) proposed the concept of “nephron dosing,” a reduction in nephrons with a fixed body mass or transplantation of a small kidney into a large recipient created an imbalance between overload and renal excretory capacity with enhanced risk of both hypertension development and chronic renal disease (Hinchliffe et al., 1991; Brenner and Chertow, 1994; Hoy et al., 2005). So, in fetal malnutrition states, the intrauterine reduction in nephron number in the full-term progeny is typically proportional to the decrease in birthweight. At birth, then, there is no imbalance between body size and nephron number.

By the way, authors have proposed, in the context of congenital reduced nephrons, the superimposition of postnatal body overweight is required to create the imbalance that generates systemic hypertension.

Emerging data now support the view that nutritional programming actively induces a propensity to accelerated postnatal growth via enhanced appetite (Vickers et al., 2000). Altered energy metabolism due to nutrient deprivation *in utero* was first described by Hales and Barker (1992) as the “thrifty phenotype.” The thrifty phenotype depicts enhanced energy utilization efficiency and insulin resistance as effective fetal strategies for preferentially shunting fuel away from muscle to protect the heart and brain. More recently, studies of fetal undernutrition have also shown the increased appetite and enhanced deposition of fat than lean tissue. Vickers et al. (2000) in a rat model of severe maternal caloric restriction throughout pregnancy, found increased food intake in offspring well into adulthood, accompanied by central obesity, arterial hypertension, and insulin resistance.

As mentioned above, prior studies confirmed a marked and sustained rise in arterial blood pressure associated with a decreased urinary sodium excretion in low-birth-weight LP compared with age-matched NP offspring (Mesquita et al., 2010a,b). However, unlike previous findings in the gestational protein-restricted progeny which demonstrated an enhanced arterial pressure beyond 7 weeks of age in LP relative to NP counterparts, a recent study in our Lab, extending the protein restriction throughout lactation, revealed a consistent onset delay hypertension in adulthood. Surprisingly, the reduced glomeruli number in this model, including protein intake reduction and breastfeeding, was higher than observed previously. A 36% reduction in nephron number was confirmed in the gestational and breastfeeding protein-restricted model compared to a 28% reduction when restriction occurred only during the gestational period (Mesquita et al., 2010a,b; Sene et al., 2018, 2021). Additionally, the expressive decrease in nephron number in the whole gestational and lactation protein-restricted model was associated with a pronounced decline in fractional sodium excretion was observed in the 24-week-old male offspring. This effect was accompanied by intensive sodium reabsorption in the proximal segments of the nephron despite unchanged creatinine clearance, potassium, and post-proximal sodium handle (Mesquita et al., 2010a,b). Herein, the reduced remaining glomerular number was associated with an increased glomerular volume, probably by enhancing glomerular volume with compensatory enhanced blood flow and glomerular hyperfiltration despite a loss of efficiency on the filter barrier (Brenner et al., 1998; Mesquita et al., 2010a,b; Pinhal et al., 2013; Sene et al., 2013, 2018, 2021; Vaccari et al., 2015; Lamana et al., 2021). Thus, the prolonged elevated glomerular filtration and blood flow processes may cause the reduced nephron number manifested as an accelerated renal function loss and glomerulosclerosis. Glomeruli overflow may be an initial insult that initiates a cascade of events, including an early inflammatory phase followed by a fibrotic response (Saleh et al., 2015). We hypothesize that the hemodynamic glomerular overload in the LP progeny compared with the NP progeny was associated with an enhanced blood flow in the remaining nephrons stimulated fibrous processes by enhancing TGF-β1 collagen 1. In parallel, an altered cellular corpuscles phenotype to epithelial-mesenchymal transition (EMT) occurred by expressing mesenchymal markers, namely, nestin and desmin. Thus, earlier findings indicated that glomerular podocytes might undergo phenotypic conversion, characterized by the loss of podocyte-specific markers and gain of transitional features, a process reminiscent of EMT (Liu, 2004, 2010; Li et al., 2008; Wang et al., 2010, 2011; Lamana et al., 2021). TGF-β triggers EMT, and its expression is upregulated in virtually every type of chronic kidney disease (Yang and Liu, 2001; Bottinger and Bitzer, 2002; Wang et al., 2010, 2011; Lamana et al., 2021), including in the diseased LP programmed model.

Although reduced nephron number may be capable of conferring vulnerability to arterial hypertension development, it clearly does not always do so. Thus, congenital reduced nephron number in humans, e.g., renal agenesis, carries a substantial risk of later hypertension and renal disease (Mei-Zahav et al., 2001). Remarkably, however, uninephrectomy in adult renal transplant donors has a very low risk of developing arterial hypertension or increased urinary protein excretion, even after decades of follow-up (Gossmann et al., 2005). The differing capacity of the renal adaptation to reduced nephron number may be critical. Animal studies support this difference as an age-dependent manifestation (Woods, 1999; Moritz et al., 2002). Relative to the adult, the immature mammal has increased renal growth potential for both hypertrophy and hyperplasia (Kaufman et al., 1975; Mulroney et al., 1999) and an increased basal level of renin-Angiotensin II (AngII) activity (Duggan et al., 1992; Kruger et al., 1998; McMullen et al., 2004). In response to nephron deficit, compensatory hyperfiltration is substantially more effective in the young than in the mature kidney (Chou and Hsu, 1991). A more vigorous compensatory tubular hypertrophy and a greater AngII response in youth may proportionally enhance systemic hypertension and chronic renal disease risk in the long term because it more effectively preserves the glomerular filtration rate (GFR). Experimental studies support the hypothesis that reduced urinary sodium excretion in LP offspring is accompanied by deregulation of sodium transporters in different nephron segments (Bertram et al., 2001; Manning et al., 2002; De Souza et al., 2004; Alwasel and Ashton, 2009). Recent data have demonstrated that a decreased urinary sodium excretion in male LP offspring was associated with sodium reabsorption in the basolateral membrane of the proximal segments of the nephron. This effect was accompanied by an increased Na/K-ATPase tubular immunomarker than the standard diet intake offspring (Fekete et al., 2008; Mesquita et al., 2010a,b; Lamana et al., 2021).

There is evidence that primary programming of the renin-angiotensin system components may be induced by a nutrient deficit *in utero* and manifested by enhanced activity postnatally, contributing to hypertension development and renal failure risk. Many studies in experimental animal models report prenatal or postnatal abnormalities of renal renin-angiotensin parameters in progeny following fetal nutrient restriction, e.g., low intrarenal AngII levels at birth (Woods et al., 2001) and increased renal type 1 AngII receptors (Whorwood et al., 2001; Vehaskari et al., 2004; Sahajpal and Ashton, 2005). The nutrient deficit *in utero* could, for example, amplify the intrarenal AngII response when body weight begins to increase. Franco et al. (2003) have reported in rats that intrauterine nutrient deficits (50% maternal calorie restriction) in 4-month-old offspring lead to enhanced AngII-induced oxygen free radical formation with the oxygen free radical formation blocked by inhibitors of NADPH oxidase. It is, therefore, possible that fetal nutritional deficit operates via a third prohypertensive pathway. Thus, direct prenatal programming of renin-AngII components, leading to postnatal enhanced AngII production amplified AngII responses and accelerated AngII-dependent injury via oxidative pathways. However, these speculative states must be confirmed considering that prior data showing that type 2 angiotensin II receptor (AT2R) is an effective inhibitor of Na/K-ATPase (Bertram et al., 2001; De Souza et al., 2004; Fekete et al., 2008). These studies have demonstrated a downregulated AT2R in adult LP male progeny, which might explain the increased Na/K-ATPase in this model (Mesquita et al., 2010a,b; Lamana et al., 2021). So, a study from Fekete et al. (2008) must be considered, demonstrating that AngII promotes Na/K-ATPase-α1 subunit translocation from the cytoskeletal fraction into the cytosol undernutrition experimental conditions.

Renal cells, including podocytes, endothelial cells, mesangial cells, and tubular epithelial cells, secrete IL-6. IL6 over-reactivity may induce mesangial dysfunction, increase cell proliferation, increase mesangial matrix deposition, and glomerular sclerosis (Coletta and Polentarutti, 2000; Gohda et al., 2001; Lu, 2008; Nechemia-Arbely et al., 2008; Ranganathan et al., 2013; Su et al., 2017). Studies suggested that fetal programming and chronic kidney disease in adult offspring are associated with increased maternal ROS production and high oxidative stress in fetal tissues (Halliwell, 2013; de Brito Alves et al., 2016). However, we observed an unprecedented increase of 32% in the NOS1 content in the renal of adult LP, relating to the kidney tubular O2 consumption in parallel with increased sodium and water reabsorption stimulation in the proximal tubule. This effect, particularly in the proximal segments of nephrons, is associated with a determining factor, which was the basolateral expression of the Na/K-ATPase pump. The elevated Na/K-ATPase in the proximal tubules of the LP offspring may involve an increase in NOS1 expression associated with increased reabsorption of sodium and water and arterial hypertension. After birth, the overload of an economic kidney may result in an increased glomerular overflow. This overload in fetal-programmed rats can result in pronounced structural glomerular disorders and accentuated and advanced stage of fibrosis promoted by TGF-β1 action inducing ZEB 2 expression, which may cause premature nephron senescence in parallel with functional loss (Lamana et al., 2021). So, it is plausible to deduce an association between accentuated reduction of the nephron numbers, decreased natriuresis, and reciprocal changes in tubular Na/K-ATPase with the development of arterial hypertension found in severe gestational and breastfeeding protein-restricted progeny compared with age-matched NP rats. Thus, recent studies observed that nephrogenesis requires a delicate balance of many factors that can be disturbed by IUGR, leading to a low nephron (Bassan et al., 2000; Sanders et al., 2000; Jones et al., 2001; Lampl et al., 2002; Hughson et al., 2003; Gopalakrishnan et al., 2005). Also, recently, in LP progeny, it has been demonstrated that reduced urinary sodium excretion is significantly attenuated by renal denervation (Custódio et al., 2017). However, studies continue to seek evidence to explain this pressure elevation, mainly implying functional kidney disorders. We may precisely suppose that increased activation of sympathetic nerves in the kidney would be associated with peripheral enhancement of the sympathetic efferent renal activity or by reduced afferent (sensory) renal nerve response (DiBona and Kopp, 1997; DiBona, 2002, 2005; Custódio et al., 2017; Kopp, 2018).

## Role of Sympathetic Renal Nerve Activity on Renal Sodium and Arterial Pressure Control

The neural activity maintains the long-term control of the structures of the different segments of nephrons and its functions through the renal efferent sympathetic nerves and afferent sensory nerves and, in this way, directly modulating arterial blood pressure. The efferent renal nerves innervate specific kidney structures, such as the juxtaglomerular apparatus, tubular cells, and renal vessels (DiBona and Kopp, 1997; DiBona, 2002, 2005; Custódio et al., 2017). This action occurs by briefly open calcium channels after arriving neural impulse at the synaptic neuron-end bulb, allowing ion influx and synaptic vesicle and membranes fusion followed by catecholamines release and diffusion post-synaptic terminals. The modulated role of renal sympathetic nerve activity (RSNA) on renal hemodynamics and tubule sodium handle has been demonstrating experimentally in heart failure and high blood pressure models. The sympathetic control of the kidney is confirmed in studies in animal models submitted to the bilateral renal denervation when the catecholamine tissue content is 95% depleted. Adrenergic depletion promotes an enhanced urine flow and urinary sodium excretion, consequently reducing arterial blood pressure (Bello-Reuss et al., 1975; DiBona and Kopp, 1997; DiBona, 2002, 2005; Boer et al., 2005; Custódio et al., 2017). Prior studies established the intimate participation of RSNA on renal dysfunctions linked to arterial hypertension development. The functional kidney effects are demonstrated by the intensity-dependent stimuli of RSNA associated successively, with a decrease in sodium excretion in the proximal tubule, enhanced renin-angiotensin system activity, decreased blood flow and GFR, and increase the resistance of renal arterial vessels (Bello-Reuss et al., 1975, 1976; DiBona and Kopp, 1997; DiBona, 2002, 2005; Boer et al., 2005; Custódio et al., 2017; Kopp, 2018). As stated above, the different glomerular and tubule functions affected by the increase of RSNA are controlled mainly by adrenergic receptors. In addition, studies have shown that the resulted renal vasoconstriction and tubule anti-natriuretic, which resulted in enhanced RSNA, is mediated through α1A- and α1B-adrenoreceptors activation, respectively (DiBona and Kopp, 1997; DiBona, 2002, 2005; Kopp, 2018). Also, at least partially, these effects may occur by α1-adrenoreceptors basolateral membrane stimulated Na/K-ATPase in proximal tubules, directly affecting sodium and water reabsorption (Aperia et al., 1992). Moreover, RSNA enables α1-adrenoreceptors to modulate the afferent and efferent glomerular arteriole resistances and change renal blood flow with repercussions on glomeruli and tubular functions (Wright and Harding, 1992; DiBona and Kopp, 1997; DiBona, 2002, 2005; Kopp, 2018).

Prior studies assume that different central nervous system (CNS) areas control the efferent renal nerve activity (ERNA) contributing to renal function control. In particular, the paraventricular nucleus (PVN) of the hypothalamus and brainstem neurons from the rostral ventrolateral medulla (RVLM) project to the kidney to regulate the renal sympathetic activity (Koba et al., 2018). Classically, the renin-angiotensin system (RAS) promotes the long-term control of water and salt homeostasis, renal function, and arterial pressure through the generation of circulating AngII. In turn, the AngII acts as a mediator of RSNA modulating sympathetic or non-sympathetic areas of the CNS (Yoshimoto et al., 2010). The type 1 (AT1R) and type 2 (AT2R) AngII receptors are distributed in several regions of the CNS (Wright and Harding, 1992). The neurons from these regions project axons to the preganglionic nucleus of the intermediolateral column (IML) in the spinal cord, which goes through the dorsal root ganglia (DRG), reaching the above-mentioned renal structures (Schramm et al., 1993; DiBona and Kopp, 1997; DiBona, 2002, 2005; Koba et al., 2018; Kopp, 2018). Evidence has identified AngII receptors expression in rat CNS, mainly AT1R, in neurons of circumventricular organs, as a subfornical organ (SFO), in the PVN and brainstem nuclei RVLM (Bunnemann et al., 1992; Song et al., 1992). This data reiterates that AngII in these central regions could drive the sympathetic fibers and RSNA. While the direct action of circulating AngII on the kidney results in sodium retention and increase of arterial pressure, the intracerebroventricular (ICV) administration of AngII conversely induces accentuated urinary sodium excretion (Wright and Harding, 1992; Schramm et al., 1993; Zapparoli et al., 2011). These studies suggest that this natriuretic effect is partly due to reducing efferent nerve and renin secretion.

Studies over the years have demonstrated that the administration of sympathetic agonists in several regions of the CNS, including the septal area, lateral hypothalamus, SFO, and anterior region of the third ventricle, lead to increased renal sodium excretion and elevated arterial blood pressure (Camargo et al., 1976; Brody et al., 1980; Bealer, 1983; Gontijo et al., 1992). Further, these studies have been proposed that effects are due to a different stimulation or receptor type expression of the α-adrenoceptors in which central α1-adrenoreceptors stimulation promotes an enhancement. In contrast, α2-adrenoreceptors have an inhibitory effect on renal sodium excretion (Koepke et al., 1988; Kapusta et al., 1989, 2012; Lutaif et al., 2015). This effect on natriuresis is intimately associated with reduced sympathetic renal neural activity (Camargo et al., 1976; Gontijo et al., 1992). The relationship between the activity and pathophysiological situations of these receptors was identified using spontaneously hypertensive rats from Kyoto (SHR) model. The centrally α1-adrenoreceptors stimulation-induced natriuretic response was blunted in SHR animals. This effect was reverted by simultaneous epinephrine and Yohimbine, an α2-adrenoreceptors antagonist, and intracerebroventricular or lateral hypothalamic area microinjection, resulting in increased urinary sodium excretion role of α2-adrenoreceptors on the hypertension development in this genetic model (Koepke et al., 1988; Kapusta et al., 1989, 2012). On the other hand, the afferent renal nerve activity (ARNA) is mediated by renal sensory neurons, located in T10–L3 ipsilateral DRG, which projects axons, almost mainly, to the wall of the renal pelvis (Ferguson and Bell, 1988; Weiss and Chowdhury, 1998). Studies have evidenced that terminal axonal neurons are activated by mechano and chemoreceptors and thermal kidney receptor stimuli (Gontijo and Kopp, 1994; Gontijo et al., 1999; DiBona and Kopp, 1997; Lutaif et al., 2015; Custódio et al., 2017). These sensory neuron ends are triggered by increased renal pelvis pressure, pelvic perfusion with different sodium and potassium concentrations, and body central temperature changes. The afferent mechano and chemoreceptors stimuli immediately cause an increased ipsilateral ARNA accompanied by decreases in the sympathetic ERNA, leading to transient but intense urinary sodium excretion and enhanced diuresis, which characterize the reno-renal reflex (Kopp et al., 1984; Gontijo and Kopp, 1994; DiBona and Kopp, 1997; Gontijo et al., 1999; DiBona, 2005; Lutaif et al., 2015). Having been assumed that the renorenal reflex is impaired during arterial hypertension development, studies in spontaneously hypertensive rats (SHR) demonstrated that renal pelvis pressure or pelvic osmotic perfusion failed to increase sensory ARNA, decrease renal sympathetic activity, and promote enhanced sodium excretion by renorenal reflex (Kopp et al., 1984, 1987, 1996; Kopp and Buckley-Bleiler, 1989; Kopp and Smith, 1991a,b, 1993; Ma et al., 2002a).

The sensory neurons in the renal pelvic are non-adrenergic and non-cholinergic mediated. Prior Immunochemistry studies have mainly localized substance P (SP) and calcitonin gene-related peptide (CGRP) in pelvic wall axonal-ends. The pelvic function of this neuropeptide was previously evaluated by studies demonstrating that the renal pelvis perfusion with substance P could activate the same afferent renal nerve fibers that are started by an increase of renal pelvis pressure (Kopp et al., 1984, 1987, 1996; Kopp and Buckley-Bleiler, 1989; Kopp and Smith, 1991a,b, 1993; Gontijo and Kopp, 1994, 1999; Ma et al., 2002a). CGRP is co-localized with SP in renal afferent sensory neurons. Also, in response to the rise of renal pelvis pressure, these neurons releases SP, and several studies have supported that SP is essential to ARNA through activation of mechanosensitive neurons (Kopp et al., 1984, 1987, 1996; Su et al., 1986; Kopp and Buckley-Bleiler, 1989; Kopp and Smith, 1991a,b, 1993; Gontijo and Kopp, 1994; Gontijo et al., 1999; Ma et al., 2002a). Through retrograde dye tracing techniques, about 90% of neurons in T10–L3 of DRG that achieve the renal hilus are CGRP immunoreactive (Su et al., 1986; Gontijo and Kopp, 1994; Gontijo et al., 1999).

However, in contrast to SP, the CGRP-mediated effect on ARNA is not entirely clear. A study has emerged suggesting that while the increase ARNA by renal pelvis mechano or chemistry stimuli is unaffected by CGRP antagonists, it seems that CGRP could hinder the SP catabolism, thereby prolonging its effects on ARNA (Gontijo and Kopp, 1994; Gontijo et al., 1999). Also, it has been suggesting that the CGRP regulates the expression of the tachykinin receptor NK1R. This G-protein-coupled receptor is the SP target of interaction in spinal cord neurons (Hershey and Krause, 1990; Seybold et al., 2003; Pennefather et al., 2004). NK1R is widely distributed in the renal pelvis, mediating the activation of renal sensory receptors in response to an increase of renal pelvis pressure (Kopp and Smith, 1991a,b, 1993; Kopp et al., 1997). Thus, because the SP, CGRP, and NK1R play a role in promoting ARNA in response to alterations in renal pelvis pressure, changes in the content of these neuropeptides in dorsal root ganglia (DRG) neurons could modulate the ARNA and renorenal reflex. As already mentioned, the renorenal reflex in SHR rats is impaired. A study conducted by our group suggests that the impaired activation of renal sensory neurons of SHR rats may be due to the changes in the expression of neuropeptides and to a decreased NK1R content in DRG neurons (Aline Boer et al., 2005). In this case, a compensatory natriuretic response does not occur to efferent sympathetic suppression response, maintaining elevated arterial blood pressure. Taking this into account, significant attenuation of tubular sodium retention and systemic hypertension after kidney denervation in maternal protein-restricted offspring is essential to assess the CNS and peripheral nerves involved in kidney function and systemic blood pressure control in programmed fetal progeny induced by severe protein restriction in rats.

## Effects of Gestational Protein Restriction on Renal Sympathetic Nerve Activity

As stated above, environmental and genetic factors may influence embryonic and fetal development, potentially leading to abnormal functional and structural effects in tissues and organs. Gestational protein restriction is associated with low birth weight and increased risk for developing cardiovascular diseases, kidney dysfunction, and metabolic syndrome in adult life (McCance, 1962; Barker et al., 1989; Barker, 1990, 1998; Nelson et al., 1998; Huxley et al., 2000). The sympathetic nervous system contributes closely to the onset and pathogenesis of arterial hypertension (Guyenet, 2006; Grassi et al., 2015; Joyner and Limberg, 2016). Specifically, renal innervation plays an essential role in developing systemic hypertension in various models of experimental hypertension. Recently, clinical trials have shown that ablation of renal nerves (renal denervation) lowers arterial pressure in some drug-resistant patients (Jacob et al., 2003; Foss et al., 2013, 2015; Osborn and Foss, 2017). The mechanisms underlying the antihypertensive effect of renal denervation are not entirely known. One important point that remains to be clarified is whether the impact of renal denervation is due to ablation of afferent [sensory, afferent renal nerve (ARN)] or efferent renal nerves (sympathetic, ERN) (DiBona and Esler, 2010; Gulati et al., 2016).

Ablation of sympathoexcitatory afferent renal nerves could, in theory, decrease arterial pressure secondary to reductions in the sympathetic drive to the kidney and other organs. Alternatively, ablation of efferent renal nerves could, in theory, reduce renal vascular resistance, renin release, and renal sodium and water reabsorption (Gulati et al., 2016; Osborn and Foss, 2017). While both hypotheses are logical, a definitive answer remains elusive. To this point, authors have shown in rodent models that renal denervation attenuates arterial hypertension in both the deoxycorticosterone acetate (DOCA)-salt and Dahl Salt-Sensitive (DS) (Banek et al., 2006; Foss et al., 2016). However, whereas this response was wholly due to ablation of renal afferent nerves in the DOCA-salt rat, the opposite was confirmed in the DS rat since selective afferent renal nerve ablation did not affect this model of salt-sensitive hypertension (Banek et al., 2006; Foss et al., 2016). These findings speak to the complexity of renal innervation action as an effective mechanism for arterial hypertension development. In addition, it was highlighting the importance of a mechanical involvement of the neural contribution of the kidney to the pathophysiological onset of arterial hypertension increase. In addition, recent studies have demonstrated that inflammatory or immune systems contribute to the pathogenesis of arterial hypertension. In this way, inflammation of the vasculature, brain, and kidneys contributes to chronic increases in arterial pressure (Marvar et al., 2010; Zubcevic et al., 2014; De Miguel et al., 2015; McMaster et al., 2015; Saleh et al., 2015).

Moreover, some reports suggest that renal inflammation may be directly caused by increased renal nerve activity. For example, Xiao et al. (2015) demonstrated that AngII-induced hypertension in mice is attenuated by renal denervation. This is accompanied by reduced renal inflammation in a pressure-independent manner (Hendel and Collister, 2006; Saleh et al., 2015). Since specific ablation of renal afferent nerves did not affect the onset and development of arterial hypertension in that study, we may suppose that the antihypertensive effect of renal denervation is mainly due to renal efferent nerve-mediated renal inflammation (Xiao et al., 2015). Similar to Xiao and colleagues studying the murine AngII model Banek et al. (2006) and (Xiao et al., 2015) have previously reported that bilateral renal denervation attenuates systemic hypertension and renal inflammation in the rat DOCA-salt model. These authors, however, did observe that resting afferent nerve discharge is elevated in DOCA-salt rats. This finding is correlated with a marked increase in several inflammatory cytokines in the kidney. In contrast to the study of Xiao and colleagues (Xiao et al., 2015), this study showed that afferent-specific renal nerve ablation attenuated systemic hypertension in this model to the same degree as total renal denervation (Banek et al., 2006). Although renal inflammation may depend on efferent renal nerves, these report data concluded that arterial hypertension was driven by increased afferent renal nerve activity, possibly secondary to renal inflammation (Banek et al., 2006; Marvar et al., 2010).

Additionally, the renal afferent nerves, comprising mechano and chemoreceptors, and temperature-sensitive fibers, originate primarily from the kidney medulla-interstitial region and pelvic wall. The renal afferent nerves contribute to sympathetic inhibitory or excitatory renorenal reflexes on efferent nerves activity, urinary sodium excretion, and blood pressure control (Lutaif et al., 2008; Johns, 2014; Kopp, 2015). Thus, the sympathoinhibitory renorenal reflex, likely driven by sensory ARN fibers, has been implicated in the tonic regulation of renal sympathetic nerve activity in healthy, normotensive animal models (Kopp, 1993, 2015; Chien et al., 2000; Ma et al., 2002a,b; Johns and Abdulla, 2013; Johns, 2014). In this way, activation of the ARN results in the suppression of efferent renal sympathetic nerve activity, promoting the renal excretion of salt and water response, thereby facilitating sodium homeostasis and the maintenance of normal pressure (Kopp, 1993, 2015; Johns and Abdulla, 2013; Johns, 2014). In contrast, the sympathoexcitatory renorenal reflex, which increases renal sympathetic outflow with sodium retention, is primarily mediated by ARN fibers that are activated in animals, including heart and renal failure models (Kopp, 1993, 2015; Johns and Abdulla, 2013; Johns, 2014; Barry and Johns, 2015). Salt sensitivity blood pressure experimental model is characterized by an exaggerated pressor response to dietary sodium intake that independently predicts hypertension risk (Franco and Oparil, 2006; Appel et al., 2011). Dietary sodium evokes a sympathoinhibitory response in salt-resistant individuals, facilitating natriuresis and normotensive response (Lohmeier et al., 1999; Johns, 2014). In contrast, sympathoexcitation promotes sodium retention and blood pressure enhancement in response to dietary sodium intake (Brooks et al., 2005; Stocker et al., 2013). It was recently postulated that neurohumoral control of tubule handle function is the first line of defense against salt-sensitive hypertension (Evans and Bie, 2016). However, the role of the ARNs in this process is unknown. A potential role for renal sensory nerve regulation of natriuresis and blood pressure is suggested by increased ARN activity during high dietary sodium intake in the salt-resistant Sprague–Dawley (SD) rat (Kopp et al., 2006, 2009, 2011). The surgical removal of sensory afferent inputs via dorsal rhizotomy or by capsaicin administration, from multiple end organs, including the ARNs, evokes salt-sensitive hypertension in SD rats (Wang et al., 1998, 2001; Kopp et al., 2003). Direct electrical stimulation of the renal afferent nerves, a non-specific stimulus that does not preferentially target mechano or chemosensitive terminals, activates hypothalamic paraventricular nucleus (PVN), parvocellular neurons, and increases blood pressure (Solano-Flores et al., 1997; Zheng and Lawson, 1997; Xu et al., 2015). These data indicate that the paraventricular nucleus (PVN) contributes to a sympathoexcitatory renoreflex. Other studies raise the possibility that the PVN could also participate in a sympathoinhibitory renorenal reflex. For example, lesions of the PVN attenuate the inhibition of renal sympathetic nerve activity associated with volume expansion (Haselton et al., 1994; Wainford and Kapusta, 2010, 2012). This suggests that the PVN integrates visceral sensory information, including the ARNs for both sympathoexcitatory and sympathoinhibitory reflexes involved in fluid and salt regulation. The results on the implications of central or peripheral sympathetic activity changes on renal function and the development of arterial hypertension in the adult rat offspring whose mothers were subjected to gestational malnutrition are preliminary, however, exciting and promising. Although the precise mechanism of these alterations is not fully understood, previous studies have raised neural mechanisms to explain most of the data found in gestational protein-restricted progeny, which involve additional alterations of RSNA in this programmed model. These studies have demonstrated that sympathetic renal nerve activity decreases renal sodium and water excretion and enhances blood pressure in the maternal protein-deprived offspring model. To addressing the neural implications of these disorders, immunohistochemistry analyses were performed. They demonstrated an increased expression of NK1 receptors and a reduced expression of the neurokinins SP and CGRP in the T13 DRGs of adult LP rats compared with NP rats. These data confirmed a significant reduction in the birth weight of LP male offspring compared with NP offspring (De Koninck and Henry, 1991; Mesquita et al., 2010a,b; Sene et al., 2013, 2021; Vaccari et al., 2015; Dasinger et al., 2016). This effect has been linked with a substantial enhancement in arterial blood pressure beyond 7 weeks of age in LP rats relative to age-matched NP counterparts. Also, the renal pelvis of LP rats did not strongly express CGRP when compared with NP rats, whereas no change was observed in SP immunostaining. These data were found in adult male LP offspring, accompanied by marked, early, and sustained (beyond 7 weeks of life) raised arterial pressure and a substantial renal decrease of urinary sodium excretion with unchanged creatinine clearance and usually filtered sodium (Mesquita et al., 2010a,b; de Lima et al., 2013; Sene et al., 2013; Scabora et al., 2015; Custódio et al., 2017; Cardoso et al., 2019). Also, a reduction in potassium excretion in LP offspring in the same studies suggest reabsorption of sodium, which may occur before distal nephron segments. The increased blood pressure and tubule sodium reabsorption are significantly blunted in LP progeny by prior bilateral renal denervation (LPDNx) compared to NP offspring (Custódio et al., 2017). Additionally, a recent study has demonstrated a unimodal distribution of neuron content SP and CGRP sub-population in NP and LP offspring, which skewed toward intermediate and large diameter cells. This skewed distribution and differences in subcellular staining showed that DRG neurons consist of various subpopulations. Particularly, significantly fewer small neurons were SP and CGRP-positive in LP offspring compared with the NP group. On the other hand, more intermediate and large neurons were observed in NP and LP offspring. The percentage of SP and CGRP-positive cells did not vary significantly between different DRG subpopulations in LP and NP rats. The precise relationship between primary afferent function and the neurochemical characterization of DRGs is still unclear. However, primary afferents of small and intermediate DRG neurons are essential for transmitting nociceptive, chemo, and mechanoreceptor information from the periphery to the CNS. They consist, respectively, of unmyelinated (*C*-conduction velocity) and thinly myelinated (*A*δ-conduction velocity) fibers, which arise from a population of cells in the sensory ganglia (Alvarez et al., 1991; Aline Boer et al., 2005). These data establish that distinct sensory neuronal populations have different functions, which may involve a differential number (small number of unmyelinated and thinly myelinated neuron cells) and expression of neurotransmitters in response to renal afferent stimuli in LP offspring. Based on these findings, the authors assume the hypothesis that an impaired renorenal reflex activity in LP offspring may be associated with a decreased expression of SP and CGRP in DRG neurons, increasing the renal retention of sodium. Defects in the level of SP receptors have been reported in non-neural vascular tissue and axonal membrane of hypertensive subjects (Huang and Koller, 1996; Schobel et al., 1996). In addition, an increased pain threshold associated with a reduction of CNS SP levels has been reported in hypertensive men and rats (Virus et al., 1981; Sitsen and De Jong, 1983; Huang and Koller, 1996; Schobel et al., 1996; Quyyumi et al., 1997). Based on these observations, we suggest that the impaired response to natriuresis associated with hypertension in LP rats could partly be related to a defect in SP and CGRP synthesis and release by renal sensory neurons. The precise mechanism for enhanced sodium retention in LP rats is still unclear. However, the above data suggested that efferent renal nerve activity and DRG neurokinin expression abnormality are conducive to excessive salt and water tubule reabsorption, which might potentiate arterial hypertension in LP progeny. Thus, these findings could raise the possibility that impaired responsiveness of renal sensory receptors in maternal protein-restricted offspring is related to altered distribution of neurokinins and their receptors in DRG neurons and, consequently, decreased concentration of SP in the renal pelvis. Also, the mechanism underlying systemic hypertension in progeny from gestational LP has not been widely identified.

**FIGURE 1:**
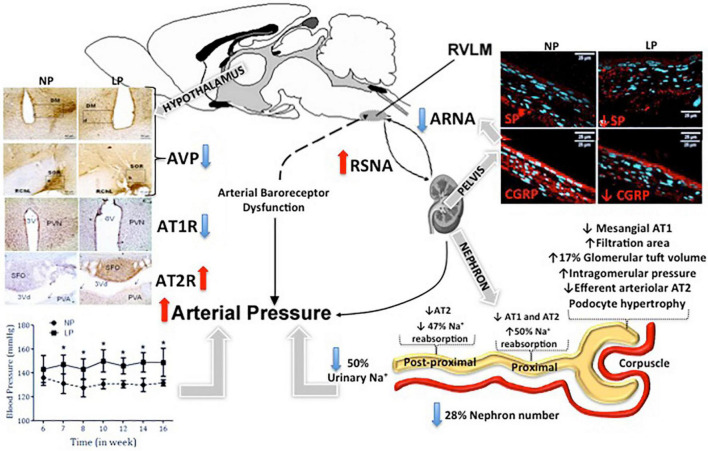
Schematic diagram of fetal programming and arterial hypertension development. The figure depicted that blunted natriuretic effect observed in low-protein intake (LP) offspring. This phenomenon may, at least in part, be due to the reduced number of nephrons associated with renorenal reflex impairment, which promoted increased renal nerve sympathetic activity, finally causing arterial pressure enhancement. This kidney neural reflex disorder may respond to the significant substance P (SP) and calcitonin-gene related peptide (CGRP) content changes of the dorsal root ganglia (DRG) in this model, which potentially participates in a deregulated afferent physiological phenomenon. Rostral ventrolateral medulla (RVLM); Renal sympathetic nerve activity (RSNA); Arginine vasopressin (AVP); Afferent renal nerve activity (ARNA); Type-1 angiotensin (AT1R) and type-2 angiotensin receptors (AT2R); Maternal normoprotein intake group (NP); Maternal hypoprotein intake group. *p < 0.05.

In part, arterial pressure is thought to be controlled by the renal-mediated regulation of fluid and electrolytes. Experimental studies support the hypothesis that fetal programming is correlated with the deregulation of sodium transporters in different segments of the nephron (Manning et al., 2002; Alwasel and Ashton, 2009; Baum, 2010). These alterations lead to a lower rate of urinary sodium excretion. Despite this, the identification of which nephron segments and sodium transporters are affected by fetal programming remains unclear. In other models of arterial hypertension described above, the bilateral renal denervation markedly attenuates the increase in arterial pressure and increased tubular sodium excretion in LP offspring. The enhanced urinary sodium excretion in LPDNx offspring was associated with a reduced proximal tubular sodium reabsorption that is incomplete, compensated by distal nephron segments. This increased urinary sodium excretion suggests an indirect but close relationship between enhanced renal nerve activity and attenuated sodium excretion in developing hypertension in LP progeny. Mizuno et al. (2013) proposed that increased blood pressure in protein-restricted offspring, in response to physical stress, would be associated with an increased renal sympathetic nerve activity (SNA), indicating a role for an increase in renal SNA in the developmental programming of increased blood pressure. Alexander et al. (2005) demonstrated that increased arterial pressure is abolished by bilateral renal denervation at 3 months of age in male and 1-year-old female offspring in the placental insufficiency rat model. These authors suggest that activation of the renal nerves is established *in utero* in male offspring. In contrast, an additional stimulus such as increased leptin plasma level in female intrauterine growth restriction offspring may be a secondary stimulus demonstrating that sex-specific programming increased blood pressure (Alexander et al., 2005; Ojeda et al., 2007; Intapad et al., 2013; Dasinger et al., 2016). Results of many studies have suggested that the SNS modulates renal function to influence the pathogenesis of arterial hypertension. Electrical stimulation of the renal nerves in acute or chronic experiments enhances tubule sodium reabsorption, particularly in the proximal convoluted tubule (DiBona, 2000a,b). In addition, studies have been demonstrated that electrical stimulation of the renal nerves at low frequency or intra-renal infusion of norepinephrine causes arterial hypertension (Collis et al., 1980; Patel et al., 1981) by increasing sodium reabsorption in the proximal tubule and the thick segment of the loop of Henle. These effects are independent of changes in renal hemodynamics (DiBona, 2000a,b). Taken together, these findings (Rudd et al., 1986; Oparil, 1987; Gerard et al., 1991; Huang and Koller, 1996; Schobel et al., 1996; Xavier et al., 2000) demonstrated that bilateral renal denervation delays the development of arterial hypertension associated with reduced sodium reabsorption by the proximal or post-proximal tubule segments in this particular experimental model of developmental programming. Although the rationale for renal denervation has generally been to interrupt sympathetic (efferent) nerve activity, the ablation of the renal plexus also deprives the sensory nerves of the kidney. As described above, selective renal afferent nerves may have widespread effects on the renorenal sympathetic reflexes and urinary sodium excretion (Kopp et al., 1987; Oparil, 1987; De Koninck and Henry, 1991; Gerard et al., 1991). Previous studies (Kopp et al., 1987; Oparil, 1987; De Koninck and Henry, 1991) have shown that increased renal pelvic pressure enhanced the release of SP and CGRP in the renal pelvis, contra-lateral urinary sodium excretion, and ipsilateral afferent renal nerve activity in rats. Additionally, studies (Kopp et al., 1987; Oparil, 1987; De Koninck and Henry, 1991; Gerard et al., 1991) from normotensive rats demonstrated that SP and CGRP elicit a similar renorenal reflex response, including an increase in renal pelvic pressure. Moreover, treatment with SP and h-CGRP8–37 receptor antagonists or capsaicin, which depletes sensory neurons of SP, blocked the ARNA response, which increased renal pelvic pressure (Kopp et al., 1987; Oparil, 1987; De Koninck and Henry, 1991; Gerard et al., 1991). However, Kopp et al. (1987) have demonstrated that increasing the renal pelvic pressure or pelvic administration of SP in spontaneously hypertensive rats failed to improve ARNA and did not elicit a contralateral renorenal reflex in these rats. The results of the current study show decreased SP and CGRP expression in T13 DRG cells in adult LP offspring compared with age-matched NP rats. Recently study demonstrated that NK1R immunoreactivity was significantly increased in the DRG cytosol and nucleus of adult LP offspring. NK1R is widely distributed in various tissues and organs. The high expression of NK1R in the sensory nervous system suggests that it plays an essential role in regulating neuronal SP and CGRP synthesis. Although NK1R expression is increased in the DRG of LP offspring, little is known about the signaling pathways that regulate the NK1 receptor gene. Since the promoter region of the NK1 receptor gene contains a cAMP response element, we hypothesize that the decreased SP and CGRP in DRG nuclei regulate the expression of NK1 receptors via a pathway involving activation of the transcription factor cAMP response element-binding protein. These findings of increased NK1R detection in DRG neurons may reflect a reduced synthesis of DRG neurokinins and explain a possible blunted renal sensory receptor activity in LP offspring. However, these observations cannot exclude the possibility that antinatriuresis observed in LP offspring could be associated with impaired neural responses to renal sensory receptor stimulation and defects in SP receptor-membrane (NK1) coupling mechanisms. Custódio et al. (2017) recently reported higher renal and plasma catecholamine levels in LP offspring compared to age-matched NP rats. Although plasmatic catecholamines may not be directly addressed as the intensity level of sympathetic neural activity in organs and systems, Custódio et al. (2017) have demonstrated that bilateral renal denervation reduced blood levels and kidney catecholamine concentrations in both NP and LP groups. These results are associated with decreased arterial blood pressure observed only in growth-restricted offspring of renal denervated control rats. Previous studies have shown that arterial hypertension associated with renal nerve activity may involve alterations in tubular sodium reabsorption, arteriolar resistance, and renin release (DiBona, 1992, 2000a,2000b; Xavier et al., 2000; Boer et al., 2005). Considering prior studies showing a peripheral sympathetic nervous overactivity, including renal nerve activity, is caused by reduced sensory renal nerve activity, recent studies suppose that the same thing may occur in LP offspring in adult life. As mentioned above, AngII plays a crucial role in the CNS areas that modulate sympathoexcitation. In adult LP offspring male rats, studies have found a reduction of AT1R/AT2R ratio expression in PVN, SFO, and solitary tract nucleus (NTS) (de Lima et al., 2013; Scabora et al., 2015; Cardoso et al., 2019). A relative decrease in AT1R was also found in the hypothalamus, kidney and heart, and other tissues in the newborn LP rats. This period preceded the hypertensive onset in the progeny in the kidneys of 1-day-old LP rats (Kannan et al., 1991). We may suppose that early-age reductions of AT1R expression may be an important causative factor for reduced sodium excretion and, consequently, hypertensive development in adult LP offspring (Figure 1). Early changes in RAS components, including Ang II receptors, could determine the optimal development and functionality of the system (Guron and Friberg, 2000). In addition, studies have been demonstrated that AngII ICV microinjection enhances urinary sodium excretion (Zapparoli et al., 2011). It is possible to argue that decreased AT1R expression in PVN, SFO, and NTS, central polysynaptic structures that maintain close connections with the sympathetic renal nerve activity, may be responsible for reducing the urinary sodium excretion in LP progeny (Gilles et al., 1999, 2001; Chen et al., 2005; Wainford and Kapusta, 2010, 2012). As stated above by studies from Xiao et al. (2015), renal inflammation may specifically be directly induced by increased renal nerve activity. Ultimately, a study in adult LP rats showed augmented Na/K-ATPase expression in proximal nephron segments, accompanied by increased NOS1 immunoreactivity in whole renal tissue. This finding was closely associated with sodium and water retention in the proximal nephron segments (Lamana et al., 2021). Lamana and colleagues also found significantly enhanced whole kidney collagen content associated with increased TGFβ1 and ZEB1/2 renal immunoreactivity in LP offspring compared with NP offspring. The expression of these increased markers in LP glomerulus was also associated with an amplified IL-6/STAT3 pathway over-reactivity that may induce mesangial dysfunction, increase cell proliferation, increase mesangial matrix deposition, and glomerular sclerosis. All these observations may provide a fresh framework for understanding the pathophysiology of glomeruli senescence and sclerosis and impaired tubular sodium handling involving multiple routes related to the development of arterial hypertension in the LP programmed model (Figure 1).

## Conclusion and Future Perspectives

Kidney function depends on the hormonal cascade, physiological mechanisms, and morphological patterns that work in synchrony. As such, any alterations in this balance can cause the onset of disease at any point in life. Though it remains to be proven, studies have shown that several environmental factors, especially exposure to a low-protein diet during critical periods of renal ontogenesis, may particularly affect the nephron numbers, surprisingly early in fetal life. Whenever nephron number is suboptimal, there are maladaptive adjustments to gene expression and long-term kidney dysfunction that may lead to cardiovascular and high blood pressure. However, it is unlikely that renal malfunction alone is responsible for to onset of arterial hypertension. Consistent data has additionally suggested that the CNS regulation of systemic blood pressure may also be altered. Taking these compiled results into account, it could be proposed that blunted natriuretic effect observed in LP offspring adulthood may, at least in part, be due to renorenal reflex impairment, which simultaneously promoted increased renal nerve sympathetic activity. This kidney neural reflex disorder may respond to significant SP and CGRP content changes in DRG in this model, which potentially participates in a deregulated sensitive (afferent) physiological phenomenon. Also, data about the central nervous system adrenergic receptor imbalance and renin-angiotensin-aldosterone system alterations may effectively change the efferent (sympathetic) renal nerve activity (Yoshimoto et al., 2010). This effect promotes sympathoexcitation in this programmed fetal model, triggering changes in renal sodium excretion and, consequently, in the arterial blood pressure control (Figure 1). The supposed centrally sympathetic overactivity in the maternal protein-restricted model, evidenced by studies mentioned above, permits to propose that other still unknown sympathetic-regulated pathways could be impaired. The elevated plasma catecholamines levels in LP progeny may result from an increased adrenal-medullar secretion, partially explained by the systemic enhanced sympathetic nerve activity. In addition, the adrenal medulla is intensely innervated (Koepke et al., 1988; Kapusta et al., 1989), which characterizes a sympathetic-adrenomedullary system (SAS). Have been hypothesized that the SAS is closely associated with the hypothalamus-pituitary-adrenal (HPA) axis activity. Both interact and promote the stress response through catecholamines and glucocorticoids adrenal secretion (Kvetnanský et al., 1993, 1995). Therefore, in the fetal programming gestational low-protein intake, there is considerable evidence of the hyperstimulation of the HPA axis and sympathetic pathways associated primarily or as a result of peripheral sensory neural disorder (de Lima et al., 2013; Lopes et al., 2013; Scabora et al., 2015; Torres et al., 2018; Cardoso et al., 2019).

Here, some pieces of evidence are gathered to establish the relevance of perinatal disorders in the origin and development of cardiovascular, metabolic, and psychiatric disorders, whose etiology is still unknown, and which clinical manifestation occurs suddenly and unexpectedly in adulthood. Learning about this theme is still sparse and grounded in experimental and epidemiological studies. This is an important area for future investigation to address the relationship and better comprehend the concurrent systems phenomenon in an integrative view in fetal programming models. Therefore, it is characterized as a broad, intriguing, and still unknown field of investigation for various areas of biological and medical fields.

## Author Contributions

VM: data curation, formal analysis, visualization, and writing – original draft. PB: conceptualization, formal analysis, resources, supervision, visualization, writing – original draft, review, and editing. JG: formal analyses, visualization, review, writing final version, and editing. All authors contributed to the article and approved the submitted version.

## Conflict of Interest

The authors declare that the research was conducted in the absence of any commercial or financial relationships that could be construed as a potential conflict of interest.

## Publisher’s Note

All claims expressed in this article are solely those of the authors and do not necessarily represent those of their affiliated organizations, or those of the publisher, the editors and the reviewers. Any product that may be evaluated in this article, or claim that may be made by its manufacturer, is not guaranteed or endorsed by the publisher.
